# Pheochromocytoma presenting as fulminant myocarditis mimicking COVID‐19 pneumonia

**DOI:** 10.1002/ccr3.5046

**Published:** 2021-11-07

**Authors:** Imen Rojbi, Meriem Adel, Meriem Affes, Saoussen Hantous, Myriam Jrad, Ibtissem Ben Nacef, Karima Khiari

**Affiliations:** ^1^ Endocrinology Charles Nicolle Hospital Tunis Tunisia; ^2^ Faculty of Medicine of Tunis University of Tunis El Manar Tunis Tunisia; ^3^ Medical Imaging Hospital of Pneumo‐Phtisiology Abderrahman Mami Ariana Tunisia; ^4^ Medical Imaging Charles Nicolle Hospital Tunis Tunisia

**Keywords:** adrenergic myocarditis, COVID‐19, fulminant myocarditis, pheochromocytoma

## Abstract

Adrenergic cardiomyopathy is uncommon but can be fulminant and life‐threatening. Nowadays, the need to exclude the possibility of COVID‐19 pneumonia in patients with acute dyspnea in a previously healthy adult may cause a delay in the diagnosis.

## Graphical abstract

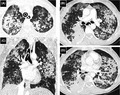

## 1 INTRODUCTION

Adrenergic myocarditis is a very rare manifestation of pheochromocytoma. However, due to the global pandemic, COVID‐19 pneumonia is now considered adifferential diagnosis of patients with dyspnea. This case illustrates the diagnostic difficulties of such uncommon disease during a pandemic.

Pheochromocytomas are rare catecholamine‐producing neuroendocrine tumors. Unusual presentations, including cardiovascular manifestations, have been reported, but fulminant myocarditis as first manifestation of pheochromocytoma is extremely rare.^1^, ^2^

Due to the pandemic situation, COVID‐19 pneumonia is now considered a differential diagnosis of patients with dyspnea. Therefore, other diseases with the same symptoms may be misdiagnosed.^3^

Here, we report a case of adrenergic cardiomyopathy mimicking COVID‐19.

## 2 CASE REPORT

A 40‐year‐old woman was admitted to the emergency department for acute shortness of breath.

Three days before being admitted, she presented with epigastralgia, vomiting, and myalgia.

On physical examination, temperature = 38°C, BP = 100/60 mm Hg, HR = 100 bpm, and SpO_2_ 100% with oxygen therapy at 15 L/min.

These findings led to the suspicion of a SARS‐CoV‐2 infection.

Blood tests showed the following results: white blood cells: 27,000/μl (normal 4000–10,000); lymphopenia: 700/µl (1000–4000); C‐reactive protein 35 mg/L (normal <5); troponin T: 8.84 ng/ml (normal < 0.05); BNP 270 pg/ml (normal < 74); and CK: 532 UI/L (normal < 171).

**FIGURE 1:**
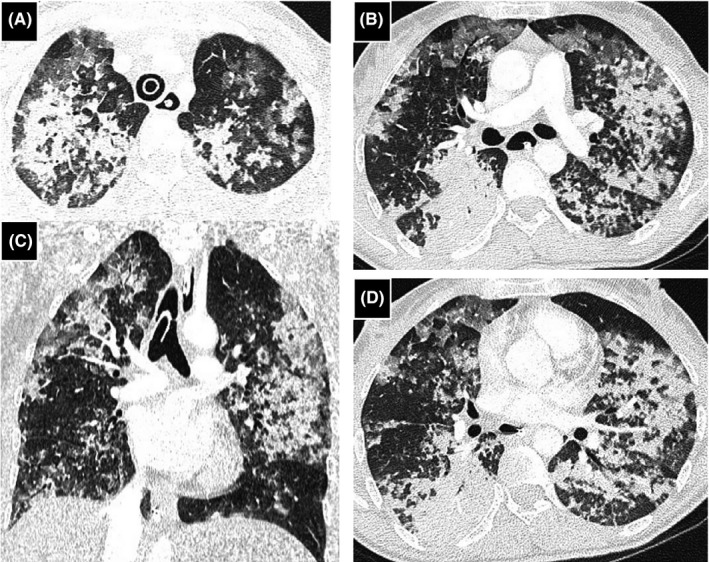
Chest computed tomography (CT) imaging, coronal (C) and axial (A, B, and D) views. Bilaterally multiple patchy ground‐glass opacities, associated with multifocal consolidations and septal thickening

The chest CT revealed asymmetric and diffuse ground‐glass opacities associated with septal thickening, consolidations, and confluent nodules (Figure 1) compatible with COVID‐19 pneumonia.

Therefore, the patient was initially treated with hydrochloroquine, ceftriaxone, azithromycine, and low‐molecular‐weight heparin.

Three COVID‐19 PCR tests were negative.

The day following her admission, the patient presented acute chest pain with tachycardia, hypotension, confusion, and cardiac arrest. The patient was successfully resuscitated, intubated, and mechanically ventilated.

The ECG showed a sinus tachycardia at 110 bpm with ST segment depression in the inferolateral leads.

Transthoracic echocardiography (TTE) revealed left ventricular septico apical hypokinesis with ejection fraction (LVEF) of 20%.

Blood examinations gave the following values: troponin: 9 ng/ml; BNP: 1558 pg/ml; and white blood cells: 27,000/μl.

Anti‐nuclear antibodies and serological tests for the most common cardiotropic viruses were negative. Multiple blood, urine, and bronchial aspirate cultures were sterile. Coronary angiography was normal.

Thus, the diagnosis of myocarditis complicated with pulmonary edema and cardiac arrest was made based on the following: hypoxemia, localized depolarization disorder, echocardiographic findings, and abnormal cardiac biomarkers.

Cardiac magnetic resonance imaging (MRI) was not done because of tachycardia and dyspnea.

**FIGURE 2:**
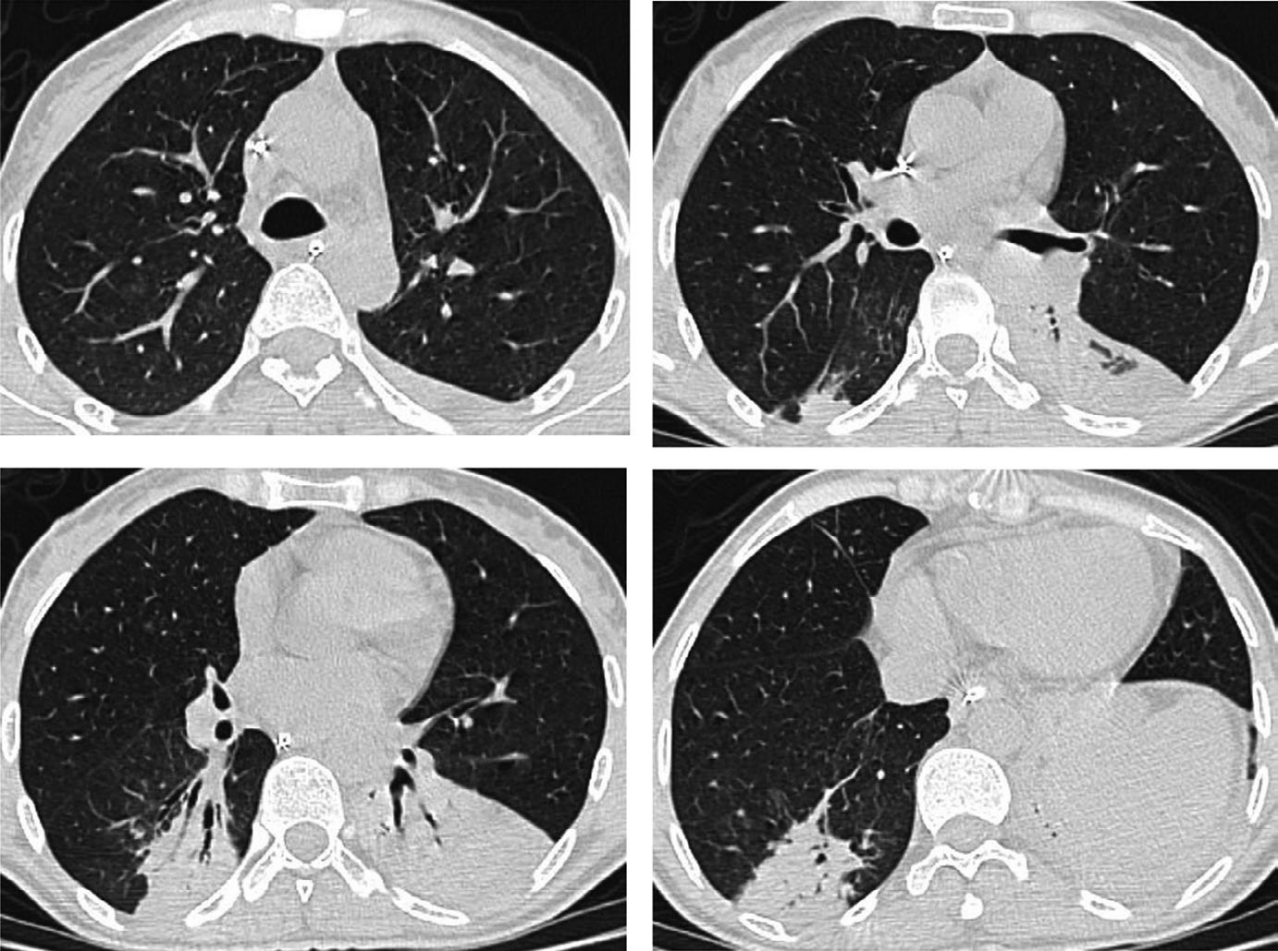
The control chest CT scan: Axial CT images have demonstrated the disappearance of previously described lesions in the upper lobes and middle lobe. It showed also a partially ventilated collapse of both lower lobes

A second chest scan (9 days after the first) showed the disappearance of the opacities (Figure 2).

SARS‐CoV‐2 pneumonia was eliminated because of a negative PCR, serologies, and the quick disappearance of the ground‐glass opacities.

**FIGURE 3:**
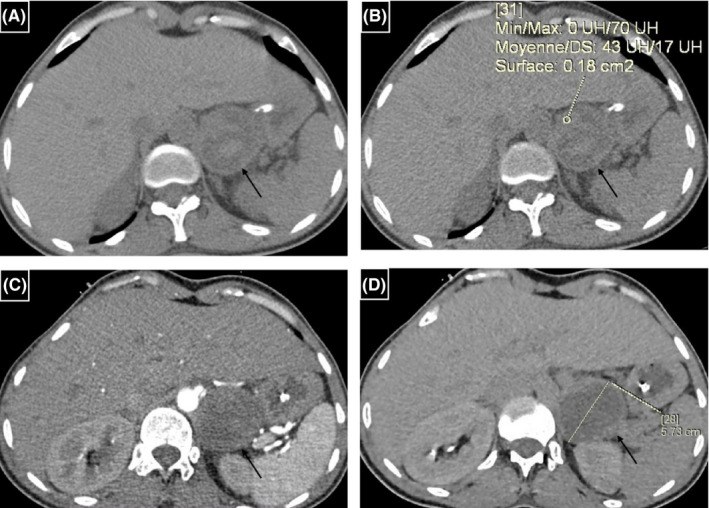
Adrenal mass on the left side (arrows): (A, B) Unenhanced CT scan contrast‐enhanced CT scans in the axial plane, (B) contrast‐enhanced CT scan (arterial phase) in the axial plane, and (D) contrast‐enhanced CT scan (delayed phase) in the axial plane. The lesion has a regular spherical shape, smooth margin, and central necrosis. Unenhanced images have shown areas of high attenuation (43HU) suggesting bleeding changes

**FIGURE 4:**
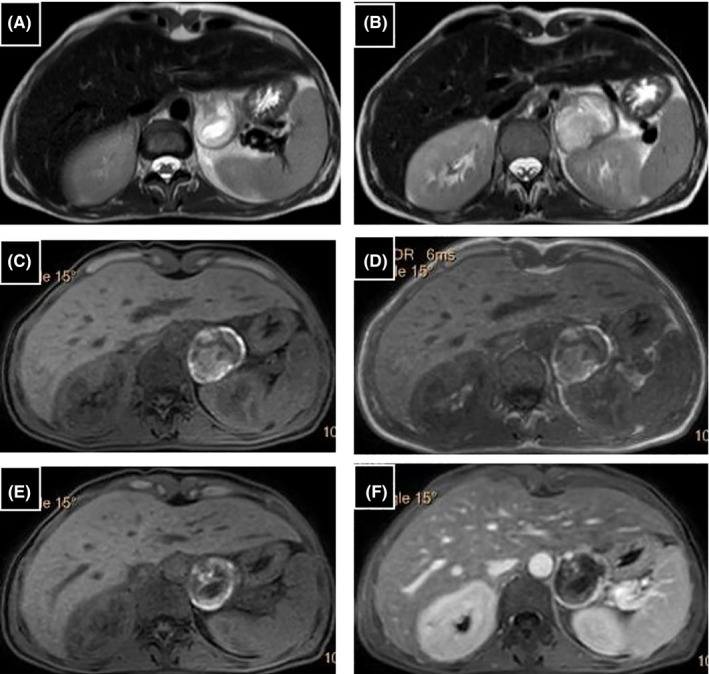
Left adrenal mass: (A, B) T2‐weighted images, (C, E) unenhanced fat‐suppressed axial T1‐weighted images have shown area of increased T1 signal intensity suggesting the hemorrhagic character of the lesion, (D) “in phase” image, (F) T1‐weighted spoiled gradient echo in the axial plane with fat saturation after contrast administration have shown of focal nodular peripheral enhancement (white arrow). T2‐weighted image showing typical high signal intensity in the central necrotic part, which appears hypointense in T1‐weighted images (black arrows)

CT slices exploring the upper abdominal floor showed a heterogeneous mass on the left adrenal gland, measuring 52 × 46 mm with a hyperdense component suggesting hemorrhagic content (Figure 3). An MRI examination showed a heterogenous adrenal mass with hypersignal areas on T1‐weighted images related to hemorrhage (Figure 4).

After stabilization, the patient was referred to the Endocrinology Department for further exploration. The 24‐hour blood pressure did not show hypertension. No headache, dyspnea, sudoresis, and palpitations were seen in the patient during admission.

Laboratory tests of 72‐hour urine catecholamines metabolites revealed mildly increased normetanephrine, but normal metanephrine was shown in Table 1. The diagnosis of pheochromocytoma was suspected.

**TABLE 1:** 72‐hours urine catecholamines metabolites

	Day 1	Day 2	Day3	Normal values
Normetanephrine nmol/creat	303	370	284	40–280
Metanephrine nmol/creat	88	86	59	15–120

TTE and cardiac MRI, performed 16 days later, were normal.

Left adrenalectomy was performed after pharmacological preparation. Histopathological evaluations confirmed the diagnosis of pheochromocytoma.

The evolution was good at the 8‐month follow‐up. No symptoms, including cardiovascular, were noted.

## 3 DISCUSSION

This case demonstrates an unusual presentation of pheochromocytoma as acute myocarditis complicated by pulmonary edema, cardiogenic shock, and cardiac arrest.

Pheochromocytoma is a rare catecholamine‐secreting tumor associated with several catecholamine‐induced cardiovascular complications.

Pheochromocytoma is symptomatic in nearly half of the patients. It is typically characterized by a triad of palpitations, headaches, and excessive sweating. Hypertension is considered the most common sign; however, about 5%–15% of patients present with normal blood pressure.^4^ Our patient was asymptomatic and had normal blood pressure.

Adrenergic myocarditis is a rare serious complication of pheochromocytoma. The mechanism of cardiomyopathy despite normal blood pressure in pheochromocytoma patients is not clearly explained in the literature.^4^, ^5^, ^6^, ^7^

Myocarditis is caused by the chronic exposure and sudden excessive release of catecholamine. In fact, long‐term elevation of adrenaline and noradrenaline leads to downregulation of β‐adrenoreceptors causing suboptimal function of myofibers and a decreased number of contracting units.

Therefore, heart failure symptoms are the common presentation of adrenergic myocarditis, making it difficult to render a definite diagnosis.^7^

It has been suggested that an acute hemorrhagic necrosis of the tumor may cause an abnormally high catecholamine release which can lead to cardiogenic shock.^4^ This finding agrees with our case in which the adrenal mass has a hemorrhagic CT density and MRI signal.

However, there has been a concern regarding the failure to diagnose COVID‐19 cases since the outbreak of the pandemic mostly due to the absence of any specific signs. This applies to viral myocarditis which has been reported in the literature as an initial cardiac complication of COVID‐19. Many patients experience tachycardia and acute‐onset heart failure with cardiogenic shock. Yet, no specific echocardiographic features of myocarditis exist.^8^

Diagnostic gold standards for myocarditis are an endomyocardial biopsy or cMRI (during the acute phase). Nevertheless, according to a position statement of the ESC diagnosis can be made from clinical and biochemical findings.^9^ In our case, the diagnosis of adrenergic myocarditis was based on clinical, laboratory, and echocardiography findings. Unfortunately, endomyocardial biopsy is not performed in our country.

The presence of fever upon admission could be explained by the cytokine release from pheochromocytoma cells.^10^

The prognosis of patients with pheochromocytoma‐associated adrenergic cardiomyopathy depends greatly on its early diagnosis.^1^ This is often challenging, despite being crucial to avoid life‐threatening complications. The current pandemic makes it even worse since it mandates the exclusion of COVID‐19 pneumonia in patients with acute dyspnea.^3^ Myocardial injuries are also common in COVID‐19 patients, accounting for 7%–23% of the reported cases in Wuha.^11^

In conclusion, adrenergic cardiomyopathy is a rare entity with a variable clinical presentation. It can be fulminant and life‐threatening. Nowadays, the need to exclude the possibility of COVID‐19 pneumonia in patients with acute dyspnea in previously healthy adults may cause a delay in the diagnosis and treatment, which increases the risks of morbidity and mortality.

## CONFLICT OF INTEREST

None.

## AUTHOR CONTRIBUTIONS

Meriem Adel involved in conception and design of study, literature search, and drafting of article. Imen Rojbi, Meriem Affes, and Ibtissem Ben Nacef involved in design of manuscript, literature search, and drafting of article. Meriem Affes, Saoussen Hantous, and Myriam Jrad involved in radiographic image acquisition and interpretation. Karima Khiari involved in design of manuscript, drafting of article, and final approval. All authors read and approved the final version of the manuscript.

## ETHICAL APPROVAL

Written informed consent was obtained from the patient.

## CONSENT

The authors have confirmed during submission that patient consent has been signed and collected in accordance with the journal's patient consent policy.

## Data Availability

The data of this case are available from the corresponding author upon reasonable request.
